# Intelligent Occlusion Stabilization Splint with Stress-Sensor System for Bruxism Diagnosis and Treatment

**DOI:** 10.3390/s20010089

**Published:** 2019-12-22

**Authors:** Jinxia Gao, Longjun Liu, Peng Gao, Yihuan Zheng, Wenxuan Hou, Junhui Wang

**Affiliations:** 1 Key Laboratory of Shaanxi Province for Craniofacial Precision Medicine Research, College of Stomatology, Xi’an Jiaotong University, Xi’an 710004, China; 2 Department of Prothodontics, College of Stomatology, Xi’an Jiaotong University, Xi’an 710004, China; 3 Institute of Artificial Intelligence and Robotics, College of Artificial Intelligence, Xi’an Jiaotong University, Xi’an 710049, China

**Keywords:** bruxism, biofeedback treatment, occlusal splint, engineering, machine learning, artificial intelligence, data analysis, stress sensor system

## Abstract

Bruxism is a masticatory muscle activity characterized by high prevalence, widespread complications, and serious consequences but without specific guidelines for its diagnosis and treatment. Although occlusal force-based biofeedback therapy is proven to be safe, effective, and with few side effects in improving bruxism, its mechanism and key technologies remain unclear. The purpose of this study was to research a real-time, quantitative, intelligent, and precise force-based biofeedback detection device based on artificial intelligence (AI) algorithms for the diagnosis and treatment of bruxism. Stress sensors were integrated and embedded into a resin-based occlusion stabilization splint by using a layering technique (sandwich method). The sensor system mainly consisted of a pressure signal acquisition module, a main control module, and a server terminal. A machine learning algorithm was leveraged for occlusal force data processing and parameter configuration. This study implemented a sensor prototype system from scratch to fully evaluate each component of the intelligent splint. Experiment results showed reasonable parameter metrics for the sensors system and demonstrated the feasibility of the proposed scheme for bruxism treatment. The intelligent occlusion stabilization splint with a stress sensor system is a promising approach to bruxism diagnosis and treatment.

## 1. Introduction

Bruxism is one of chronic dental problems worldwide with multifactorial etiology and no golden standard for diagnosis and treatment [1]. The disorder is defined as a repetitive jaw muscle activity characterized by clenching or grinding of teeth and/or by bracing or thrusting of the mandible [2]. Previous investigations found that the prevalence of sleep bruxism (SB) was about 50% in adults [3] whilst the prevalence of SB in children ranged from 3.5% to 40.6% [4]. Although signs and symptoms of bruxism vary, it is always supposed to be an etiological factor in causing damage to supporting structures of teeth, abnormal tooth wear, failure of dental restorations, and temporomandibular and musculoskeletal disorders [5].

Current diagnostic methods for bruxism mainly include self-report, clinical examination, electromyography (EMG), and polysomnography (PSG) [1]. According to an international consensus discussion summary, the existing assessment of bruxism could also be classified into three main aspects: (1) noninstrumental approaches, (2) instrumental approaches, and (3) cut-off points grading [6]. Self-report and clinical examination are considered as noninstrumental approaches, which are also the primary choices in the clinical assessment of bruxism. However, their reliability and validity need further improvement [6]. EMG may provide good evidence of both sleep and awake bruxism, but there is also a risk of overestimating the number of true SB events [7]. PSG could be regarded as a reference standard for SB assessment; however, it is expensive and time-consuming [2]. To date, the reliability and validity of all the common techniques remain debatable, and consensus has yet to be established regarding the best method to diagnose bruxism. Therefore, exploring some new methods of bruxism diagnosis and management is a necessary and meaningful research topic.

The use of biofeedback technologies (electrical, auditory, vibratory stimulus, etc.) as behavioral techniques of bruxism diagnosis and treatment has been considered a promising approach in both clinical and scientific fields in recent years [8]. Various biofeedback modalities have been reported in previous papers, and most of these are based on EMG recordings, except two studies that described force-based devices [8,9]. An intra-splint force detector (ISFD) for SB force detection was described in 2003 [10]. A detailed description of this SB inhibition system has been presented in a recently published paper [11]. This inhibition system consisted of ISFD, vibration, and control units. 

The ISFD used a modified occlusal stabilization splint (OSS) as a carrier, which was fabricated by a heat-curing resin. A 100 μm thick deformation-sensitive piezoresistive film was embedded in an oral appliance for occlusal force signal detection. Another kind of mini wireless biofeedback device for SB diagnosis and treatment was reported, in which a stress sensor, a button cell, and a mini monitoring circuit were embedded in the special splint and a watch style device was designed for monitoring and feedback [12]. Pilot studies found that biofeedback therapy based on occlusal force could monitor occlusal changes effectively [10,11,12]. 

However, the occlusal force-based detection system was mainly used in SB, and the sensors were packed only in the canines or premolars regions. The existing limitations in previous studies on this topic gave rise to several questions. According to the current concept, an OSS was characterized as conservative, reversible, and nonspecific in bruxism diagnosis and treatment [13]. An OSS had a full coverage of the maxilla or mandibular dental arch and has been proposed to balance the force distribution to the entire masticatory system [14]. Moreover, an OSS could provide an ideal occlusion and reduce abnormal muscle activity, and it can be used to protect teeth and support structures of bruxers [15]. In this regard, an OSS with a full coverage of dentition in combination with multi-site stress sensors can show better detection and protection capabilities of both sleep and awake bruxism.

In recent years, artificial intelligence (AI) algorithms (e.g., deep learning algorithms) have developed rapidly along with continuous integration with the medical field, including medical imaging, health management, early detection of diseases, biotherapy, and precision medicine. Hannun et al. [16] used AI algorithms to diagnose heart rate irregularities based on arbitrary-length, single-conductance electrocardiogram (ECG) time series data. The study used a Zio heart rate monitor to collect ECG data and perform training and detection via an AI algorithm. 

Ravizza et al. [17] used AI algorithms to leverage clinical data to predict early risk of chronic kidney disease in diabetic patients. Their AI algorithms use clinical data with a complete data volume and consistent consistency to predict a diabetes-related chronic kidney disease model better than traditional clinical research data models. AI technology has also been extensively studied in the field of medical imaging, but the research on real-time, accurate and intelligent detection of oral occlusal stress has not been reported in detail [8]. With the progress of science and technology and the emergence of medical and engineering strategies, it is now possible to design and create a portable, intelligent and precise force-based bruxism feedback detection system. 

In this study, a real-time, quantitative, intelligent biofeedback system to detect occlusal force based on an AI algorithm is proposed. The purpose of this study is to test the hypothesis that this system can analyze occlusal contact precisely and detect occlusal force change quantitatively. At the same time, this work also aims to assist doctors in monitoring bruxism by a mobile application and assessment in real time for necessary interventions. 

**Figure 1:**
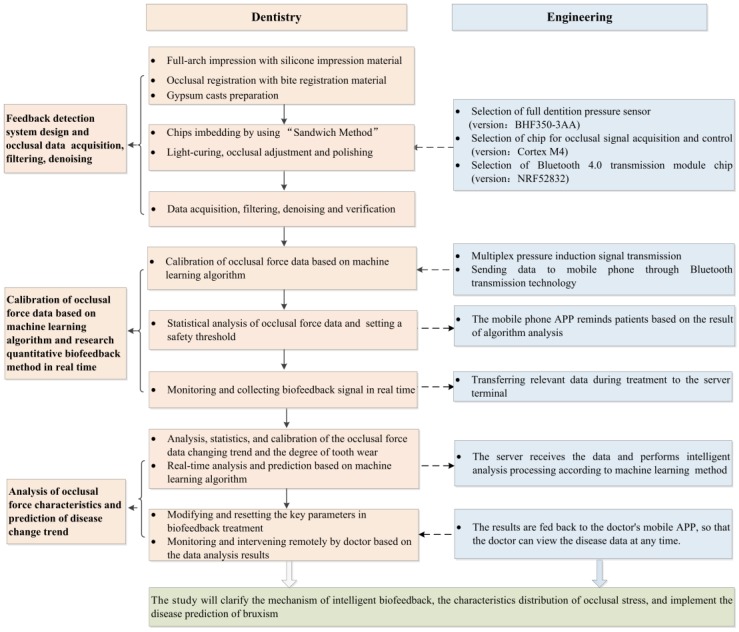
Overall illustration of the interdisciplinary research and three main contributions of this study.

This study employs an interdisciplinary approach that integrates dentistry and engineering concepts. Its main contributions study can be summarized into three aspects: (1) the proposal of a design for full-dentition, stress-sensitive occlusion detection and stress data acquisition, filtering, and denoising; (2) the calibration of occlusion data based on a machine learning algorithm and the study of a real-time quantitative biofeedback method for occlusion detection; (3) the analysis of occlusal stress characteristics and disease status prediction. The overall key points of our study are also shown in Figure 1.

The rest of this paper is organized as follows: in Section 2, materials and methods are presented in detail. The experimental results are described in Section 3. In Section 4, the sensor system and future research are discussed. At last, we conclude our study in Section 5.

## 2. Materials and Methods

### 2.1. Overall Treatment Scheme: Biofeedback System Scheme

**Figure 2:**
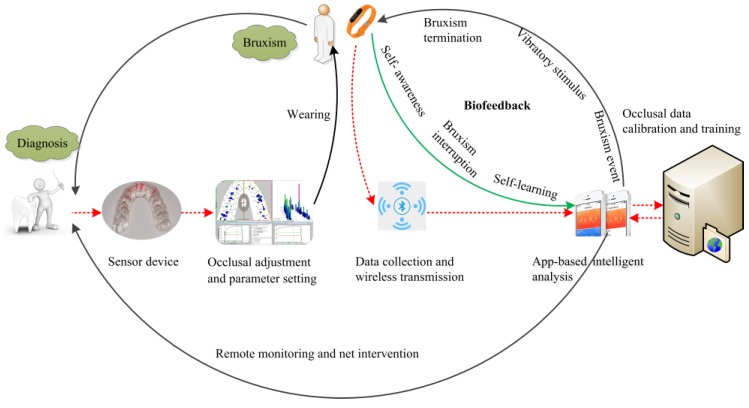
Overall treatment scheme of the biofeedback system we proposed for bruxism.

Given that biofeedback training is growing increasingly popular in recent years [18], a sensor device in this concept was designed to record bruxism behaviors. An occlusal force produced during teeth bracing and/or grinding events as input signals for bruxism was detected and transmitted effectively by embedded chips. A control chip then converted the inputted occlusal force signals into digital signals by using a digital-to-analog converter and transmitted data to a Bluetooth sending chip. Then, the Bluetooth receiving segment of a mobile phone received the signals based on the Bluetooth protocol. Wireless Bluetooth transmission was mainly used to send the occlusal force data processed by a microprogrammed control unit (MCU) to a server terminal. Abnormal and frequent occlusal force signals were converted to vibratory stimulus signals through a smart watch, and the bruxer was reminded to prompt the immediate relaxation of the jaw muscles. The biofeedback system aimed to generate a learned response and finally terminate the bruxism behavior without wearing a feedback system. In addition, occlusion time, occlusal force distribution, and occlusal contact could be displayed on the smartphone, thus providing real-time tracking for self-care. Moreover, clinicians can also share and analyze data for real-time, remote monitoring in clinical practice. The protocol of the biofeedback system is presented in Figure 2.

### 2.2. Full-Dentition Stress Sensors Integration and Imbedding Methodology—"Sandwich Method”

There are many challenges for stress sensor devices integration and imbedding in the mouth. How to successfully integrate a sensor chip into a pad and operate it normally is a key problem. A full-dentition occlusion detector needs to be worn on a patient’s upper or lower jaw dentition, so the embedded chip must be as small as possible to achieve actual embedded installation requirements; the embedded chip should be isolated from the patient’s mouth to achieve a sealed package. 

#### 2.2.1. Early-Stage Preparation

An extensive clinical examination was conducted to assess occlusal features, and a silicone bite registration material (DMG, O-Bite, Hamburg, Germany) was used to measure occlusal contact areas in the maximum intercuspal position [19]. Full-arch impression was taken by using a silicone impression material (Silagum Putty/Silagum Light, DMG, Hamburg, Germany). Gypsum casts (Heraeus, water/powder: 22 mL/100 g) were obtained from silicone impression and mounted on an articulator (Stratos300, Ivoclar Vivadent, Liechtenstein) combined with occlusal registration and face-bow transfer.

#### 2.2.2. Sandwich Method

The OSS had a coverage of an approximately 2 mm width on both the buccal and lingual surfaces to provide friction retention. The desired border was initially marked on the maxillary working cast with a red pencil. Then, an about 1 mm thick light-curing transparent resin (3M, Z350 XT, St. Paul, MN, USA) was evenly applied to cover the buccal, occlusal, and lingual surfaces of the upper full arch. All the residual resin from the working cast was removed according to the outlined finish line by using a laboratory knife, after which stress-sensitive chips and control chips (approximately 0.3 mm thick) were embedded in the marked sites of the OSS. Another layer of the transparent resin (about 1 mm thick) was evenly applied to cover the chips and the first layer of resin before light-curing (3M, Elipar™ LED Curing Light, Light curing unit power: 1200 mW/cm², Light curing time: 20 s, St. Paul, MN, USA). Finally, the occlusal adjustment of the ideal contact areas was conducted, and the device was polished. 

When considering an OSS for diagnosis and treatment of bruxism, occlusal adjustment plays an important role, and the ideal occlusal contacts should achieve the following goals. In maximal intercuspation, mandibular posterior teeth must come into contact with a device providing even force and contact slightly more heavily than anterior teeth. Posterior teeth should be separated in protrusive and lateral movements [20]. During the protrusive movement, mandibular anterior teeth must come into contact with the device providing even force. In the lateral movement, only mandibular canines should exhibit laterotrusive contact with the device [20]. As the entire force of a protrusion on any single tooth may cause occlusal trauma, any interferences must be eliminated during occlusal adjustment. 

### 2.3. Sensors Components and System Design

The sensor system mainly consisted of three parts: a pressure signal acquisition module, a main control module, and a server terminal.

- The pressure signal acquisition module included a set of sensitive piezoresistive chips (HuaLanHai, BHF350-3AA, GuangZhou, China). The sensor chip can output corresponding voltage changes with dental pressure variation.
- The main control module was mainly used for receiving occlusal force signals collected by a stress sensor, while also processing, storing and packing the collected data. In this system, a ultralow-power MCU (Nordic Semiconductor, NRF52832, Oslo, Norway) was used as the main controller to control the acquisition of multiple stress signals, convert analog signals into digital signals through a multichannel analog-to-digital converter (ADC) (Nordic Semiconductor, SAADC, Oslo, Norway) conversion module and then send the processed data to a server for further analysis. The power supply of the control module was accomplished by using a button battery (Panasonic Semiconductor, Panasonic CR2032, Celebes, Indonesia), which can work continuously for at least six months. The MCU should turn off automatically, whenever the required operating voltage could not be supplied by the battery.
- The wireless transceiver module (ST Microelectronics, STM32, Geneva, Switzerland) was mainly used to send the occlusal force data processed by the MCU to a server terminal. Bluetooth wireless transmission has the advantage of low power consumption and significant reduction of power consumption of a sensor system, which can be used to update occlusal data in real time. The server terminal parsed the data package according to the Bluetooth protocol and stored the parsed data in the server for further data analyses.
- The server terminal module utilized a common server (Hewlett-Packard Development Company, L.P, HP Z840, Beijing, China) with a Graphics Processing Unit (GPU) coprocessor (NVIDIA, TiTan XP, Santa Clara, CA, USA) for machine learning algorithm training. The server platform ran application software to receive data from the sensor system and showed the curves generated based on sensor-captured pressure.

#### 2.3.1. Sensor System Design Consideration

A whole sensor system integration solution was designed from scratch, taking care of both hardware and firmware designs. In order to achieve real-time, dynamic monitoring of bite forces, an occlusal splint must be placed inside the oral cavity, and the comfort of a patient’s wearing needs to be taken into consideration to be compatible with human tooth structures. Therefore, the entire system must have the characteristics of microsize, low power consumption, and stable performance. The main control module is the core and foundation of the entire detection system, which determines whether the entire system can detect and transmit data stably and accurately

The server mainly used a machine learning algorithm to further process bite force data and combined digital signal processing (signal filtering and smoothing) and statistical analysis to analyze signals. The statistical analysis mainly included five aspects: (1) average value (*AVGx_i_*) of bite forces in a monitoring duration, e.g., 24 hours or one week, (2) maximum (*MAXx_i_*) and minimal (*MINx_i_*) amplitude values of bite forces, (3) standard deviation (σ*x_i_*) value of bite forces during a monitoring period, (4) variance value (ψ*x_i_*) of bite forces in a monitoring duration, (5) covariance value (φ*x_i_*) of all bite forces during a monitoring period. According to these analyzed results, the algorithms can give a conclusion whether the subject has bruxism or occlusion problems (e.g., the type, the severity, and the trend of the illness).

#### 2.3.2. Workflow of an Intraoral Sensor Pressure Detection System

**Figure 3:**
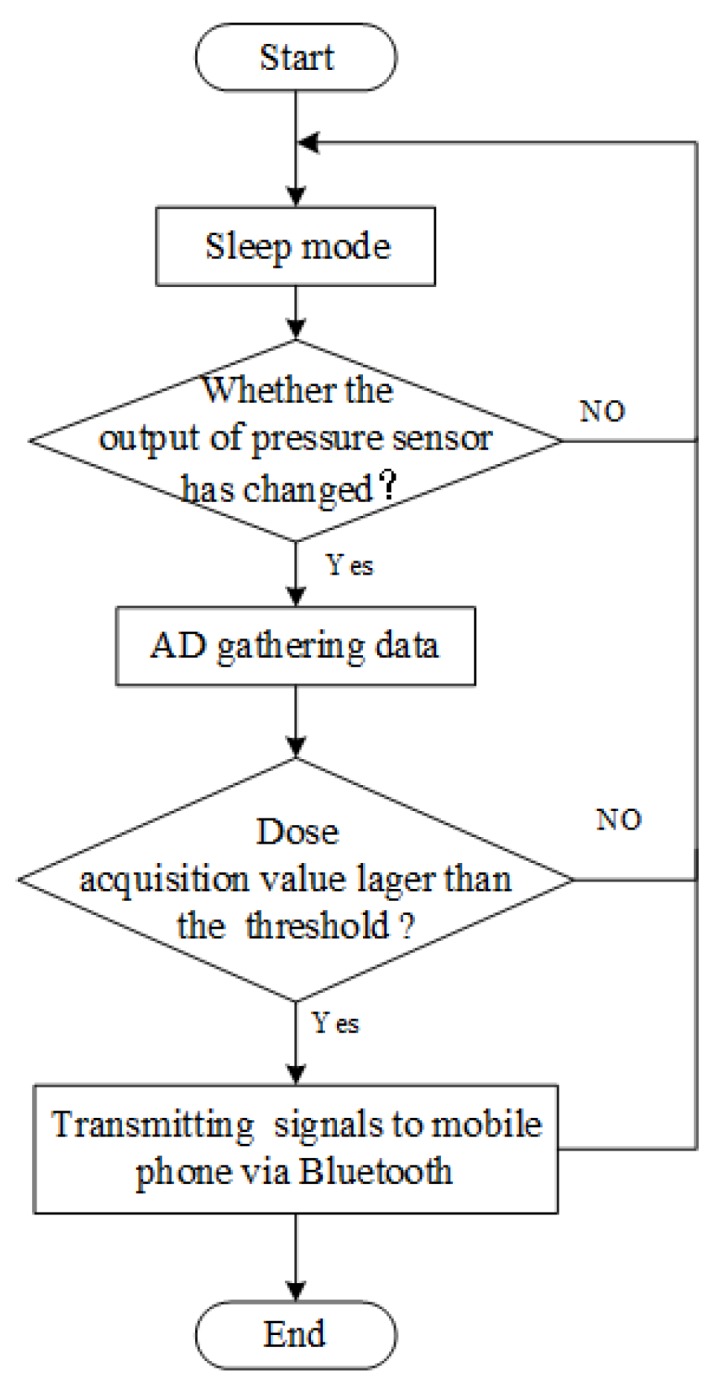
Flow chart of an intraoral sensor pressure detection system.

Initially, the sensor system is in a sleep state to reduce system power consumption. When the patient experiences occlusal contact and produces a resultant force to the stress sensor, this causes the voltage value of the sensor output to change, which is transmitted to the AD converter for data conversion. In order to prevent false positives and record the resultant force start time accurately, the occlusion detection system stores an initial threshold set by the dentist in the first appointment. During biofeedback testing, the dentist can remotely reset the threshold based on feedback analysis data. When the digital signal collected by the AD converter is greater than the threshold, the abnormal occlusion event is considered to occur. The MCU is awakened, and the multichannel AD conversion-processing port detection module is implemented, which mainly includes the following operation steps, as shown in Figure 3.

### 2.4. Data Collection and Analysis

#### 2.4.1. Data Denoising

In this study, a data signal is denoised by wavelet transform, that is, a noise- and stress-induced signal is transformed into the wavelet domain, and then the wavelet transform of the signal is separated from the wavelet transform of the noise; finally, the noise transform coefficient is discarded, along with the remaining transform coefficients. The inverse transform is used to obtain a denoising signal (relevant parameters are obtained by medical statistics experiments). First, the wavelet transform is used to transform an occlusion stress data signal into the wavelet domain, after which the wavelet transform of the signal is separated from the wavelet transform of the noise. Finally, the noise transform coefficients are discarded, and the residual transform coefficients are inversely transformed to obtain the denoising signal. 

In many wavelet transform denoising methods, the translation invariant wavelet denoising method was selected to denoise the number. In particular, if the original signal obtained is *X(t)*, where 0 *≤ t ≤ n*, and the signal *S_h_(t) = X(t + h)* is obtained by the time-domain translation of a positive integer), then under the condition that *S_h_* is reversible, the translation invariant wavelet denoising method of *n*-cycle translation can be expressed as:

$$\bar{T}(x;{\left(S_{h}\right)}_{h\in H_{n}})=Ave_{h\in H_{n}}(T(S_{h}))$$

where *H_n_ = {h:*0 *≤ t ≤ n*}, Ave means the average value, and *T(S_h_)* conducts the denosing process for signal *S* with the Donho threshold method.

#### 2.4.2. Data Analysis with Intelligent Machine Learning Algorithms

**Figure 4:**
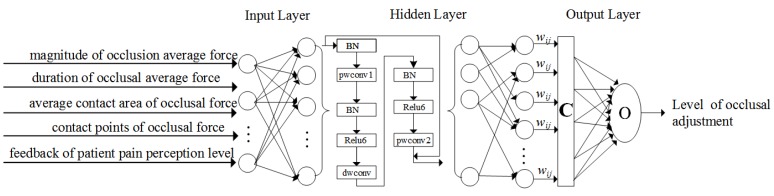
Neural network model designed in the present study.

Based on the collected occlusal force data, five important characteristic parameters in the occlusion adjustment per unit time are extracted as AI algorithm inputs: magnitude of the average occlusion force, duration of the average occlusal force, average contact area of occlusal forces, contact points of occlusal forces, and feedback of a patient pain perception level. The bite force and the force time are obtained by a sensor detector. The patient feedback sensation level refers to the pain during the occlusion process that the patient feeds to the intraoral detector through the biofeedback system. Grade is initially defined by a doctor and is determined by multiple sets of experiments in this project. The contact area and the number of occlusal contact points are obtained by conventional occlusion paper and the T-Scan method. Then, a one-dimensional deep convolutional neural network is used for intelligent analysis and auxiliary diagnosis, as shown in Figure 4. 

The algorithm uses the neural network of residual structure to improve the test accuracy of the network, where pwconv1 and pwconv2 represent one-dimensional pointwise convolutions, dwconv represents a one-dimensional depth-wise convolution, BN represents batch normalization and Relu6 represents a nonlinear activation function. The output of the neural network is the level of occlusion adjustment, as shown in Figure 4. Each level has a special altitude for occlusion adjustment, e.g., each level represents an operation that rises or decreases by 0.5 mm. The initial value of the level of occlusion adjustment is set by a dentist. Then, the neural network is trained based on the initial experimental value and the training sample. According to the abundant input data for training the sample (about larger than 50 thousands training samples), the neural network can learn to output a proper level of occlusion adjustment based on the unknown input of the neural network, which can guide the occlusion adjustment for the dentist. In this project, the occlusion adjustment was finally determined by multiple sets of experiments. Prior to the neural network algorithm training, the clinician must first calibrate the degree and level of the input data and the corresponding occlusion adjustment, so that the algorithm model first obtains the network parameters on the calibrated data set and then analyzes the actual data based on the learned model parameters. As for the test, the neural network training process used a momentum gradient descent method with a coefficient of 0.9 to train the network, and the batch size was set to 128. In addition, the migration learning method was used to pretrain the network with external data, before the training began to obtain better initialization weights.

Through training and testing experiments to improve the accuracy of the algorithm, 80 sets of the sample calibration training data were set in the specific implementation, and 20 sets of the sample inference data were tested. The neural network training data were collected from different sick periods of the same patient, thus forming a data set. The occlusion behavior and degree level identification was performed through the stress data of the patient per unit time obtained in real time to assist the doctor in diagnosis and treatment.

### 2.5. Diagnosis and Treatment Scheme

The initial occlusion detection algorithm parameters and thresholds are set by analyzing the occlusion status of different patients with the initial diagnosis. After the patients wear the monitor, their real-time occlusion stress data are collected based on the set threshold, thus providing a basis for generating biofeedback events.

The occlusion data are stored in a mobile application via the Bluetooth transmission technology. Based on the occlusion stress determination algorithm integrated in the mobile application, the patients’ real-time occlusion changes are analyzed, and the patients are sent a message according to the algorithm result. The patients are reminded to check the current occlusion stress condition, and the current pain levels are recorded on the mobile application. The mobile application records the patients’ input, and the collected data are then uploaded to the server for analysis. 

**Figure 5:**
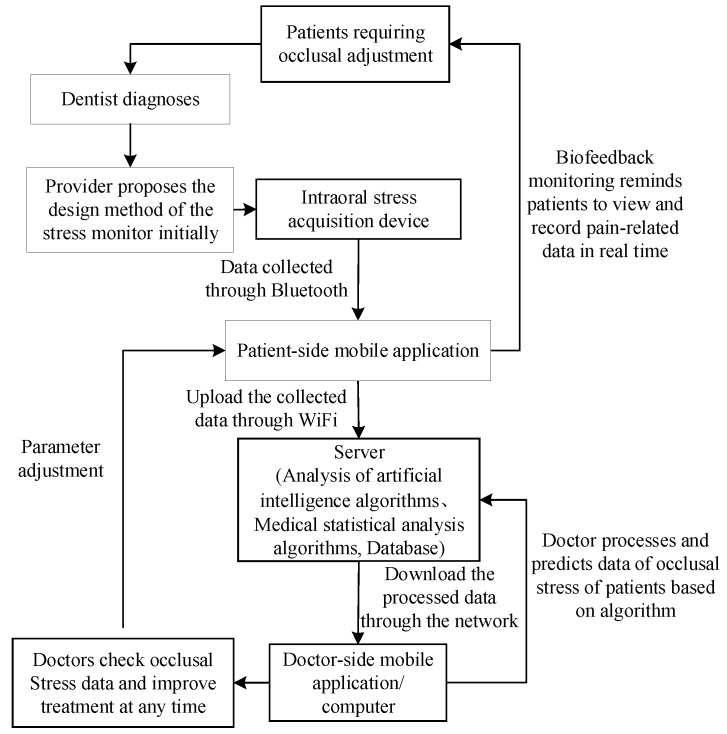
Overall diagnosis and treatment scheme for bruxism.

On the server, the patient’s occlusion analysis results are obtained by an AI algorithm. The doctor can view the occlusion stress data at any time through the doctor’s mobile application. Based on the analysis results, the parameter threshold value during the biofeedback treatment process can be adjusted remotely in real time to achieve the purpose of real-time quantitative biofeedback detection and recording, as shown in Figure 5. 

## 3. Results

In this section, we present the prototype of our sensor system. We show the experiment results with the sandwich method and the electrical measurements of the sensors; we also demonstrate the feasibility of the sensors scheme we proposed. In future experiments, our sensor system will be embedded in patients intraorally. 

### 3.1. Sandwich Method Verification

**Figure 6:**
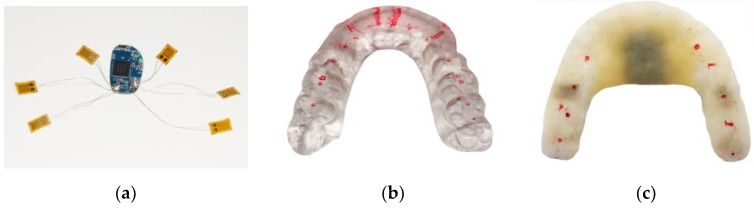
(**a**) Stress-sensitive chips and control chips; (**b**) occlusal contacts of an occlusal stabilization splint (OSS); (**c**) sensors packaged in the OSS.

**Figure 7:**
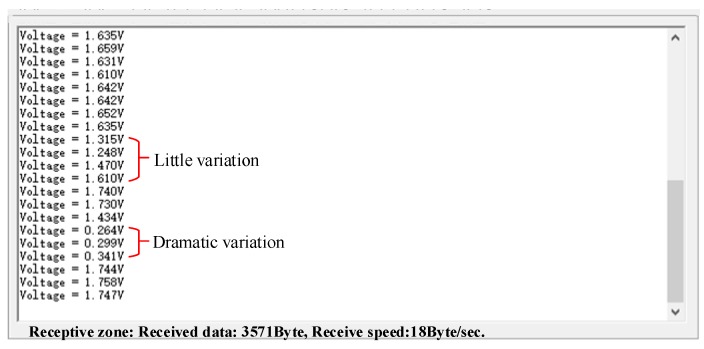
Data acquisition from stress sensor system.

We completed the sensors embedding in an OSS based on our sandwich method, as shown in Figure 6. Figure 6a presents the stress-sensitive chips and the control chips. Six sensors were used in this paper for data acquisition and bruxism diagnosis. The data collected from the six sensors were voltage signals, as shown in Figure 7. In this paper, we only present the data graph of one sensor shown in Figure 7. Figure 6b presents the preliminary production of a traditional OSS, i.e., the occlusal contacts of anterior guidance, canine protection, and maximal intercuspation after occlusal adjustment. Figure 6c shows the sensors were packaged in the OSS. We further studied the reasonable parameter setting for the sandwich method, as shown in Table 1. 

### 3.2. Sensor Prototype System Building 

**Figure 8:**
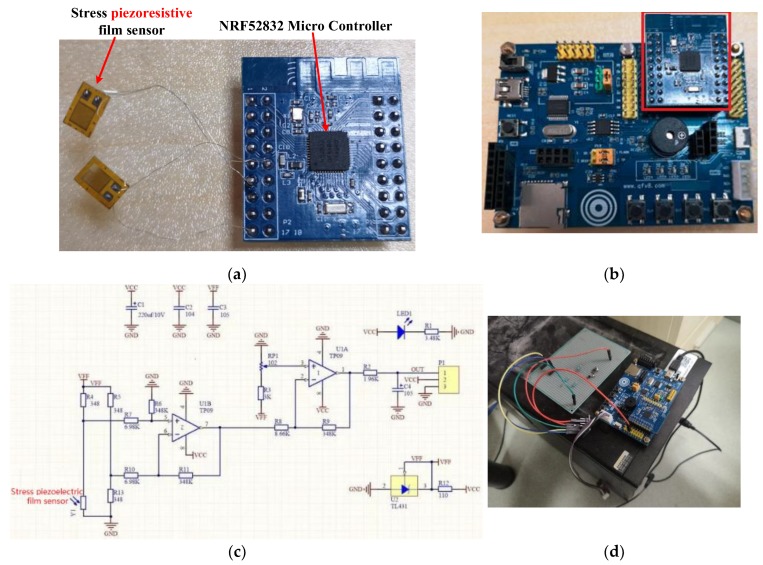
(**a**) Pressure signal acquisition module; (**b**) test board for a microcontroller circuit board (the microcontroller circuit board is indicated by a red rectangle); (**c**) electric schematic diagram of the pressure signal acquisition module; (**d**) prototype system connected with the server.

We conducted a sensor prototype system from scratch. Figure 8a shows the pressure signal acquisition module, which included a group of piezoresistive-film stress sensors and a microcontroller. The module was embedded in the OSS with our sandwich method. The current size of the module was about 10.5 mm × 8.2 mm. We further customized a circuit board and removed several test pads, when it was embedded in the OSS. In this way, the size was reduced to 6 mm × 6 mm. The button battery was stacked up on the back of the circuit board. Figure 8b shows how the microcontroller circuit board was placed in a test board. Based on the test board, we were able to fully verify the function of the pressure signal acquisition module. Figure 8c shows the electric schematic diagram of the pressure signal acquisition module. Figure 8d presents our prototype system connected to the server for real-time debugging with a serial port.

### 3.3. Experimental Data from the Sensor Prototype System

We collected the data from stress signals and data transmission and receiving processes separately. Figure 7 shows the data collected from the pressure signal acquisition module, which were also the input signals for the subsequent module of statistical analysis and neural network analysis. The input voltages in the experiment were shown, and as can be seen, there are two typical areas: little variation and dramatic variation, referring to small occlusal forces and large occlusal forces, respectively, obtained from the piezoresistive-film stress sensor. Figure 7 also demonstrates the voltage variation of strain gauges, which indirectly reflected the resistance change of strain gauges. From such data, we can obtain the severity degree of a patient’s bruxism episode. Although several noise data from the pressure signal acquisition module can be found, our denoising algorithm can effectively remove such abnormal noise data. The feedback signals in this paper are the level of occlusion adjustment from the neural network, which can be input into the biofeedback system for bruxism diagnosis. Both the input signals (Figure 7) and the feedback signals can be collected at any time, which can be demonstrated in multiple cycles.

**Figure 9:**
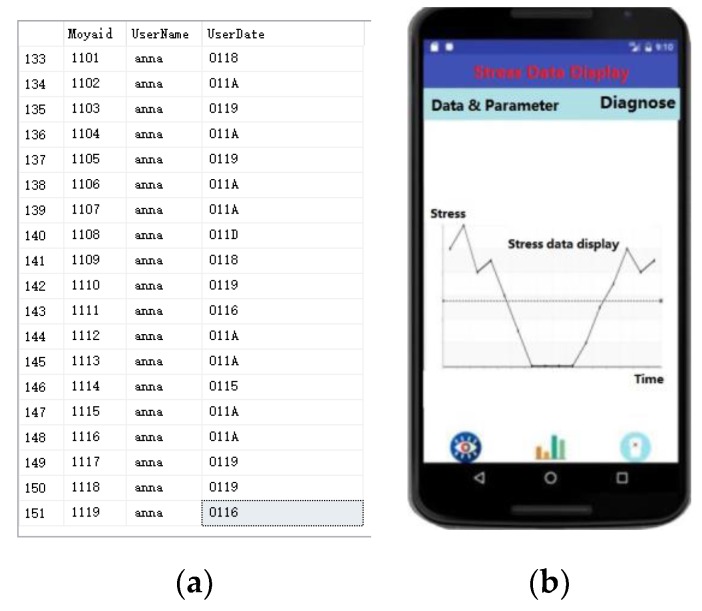
(**a**) Data stored in a table on the server; (**b**) data visualized with various curves in a mobile application.

We further tested the data transmission from the phone to the server based on the Bluetooth protocol. The data were coded for transmission based on the transferred equation: coded data = Sample_Voltage × 3.6/1024. The coded data were sent to the server and stored on the server, as shown in Figure 9a. The data can be visualized with various curves by a mobile application, so that a doctor can check a patient’s condition at any time, as shown in Figure 9b.

### 3.4. Experiment of the Configuration of a Machine Learning Algorithm

To intelligently analyze the bruxism data collected from the sensor system, we leveraged a machine learning algorithm. As is well-known, various machine learning models can be used to analyze data. In this paper, we tested many current machine learning algorithms and found that the neural network is a good choice because it features very efficient and simple data analysis for processing bruxism data. Finally, we found a useful and feasible neural network model for our system, as shown in Table 2.

## 4. Discussion

### 4.1. Main Results

The use of biofeedback for treatment of diurnal and nocturnal bruxism has been discussed comprehensively over the years [21]. The clinical use of occlusal splints for biofeedback has been reported in previous studies as well. Biofeedback by using a flat occlusal splint to remind a diurnal bruxer of abnormal tooth contacts obtained an immediate success of approximately 50% [22]. To the best of our knowledge, this is the initial attempt to research a real-time, quantitative, intelligent, and precise force-based biofeedback detection device for diagnosis and treatment of bruxism. The study found that a full-dentition stress sensor will be a promising device in monitoring changes of a patient’s bracing or grinding and recalling self-awareness to behaviors effectively in future practice.

### 4.2. Occlusal Adjustment

The advantages and limitations of using the modified new device are discussed in this subsection. Accurate occlusal adjustment is essential in achieving multiple contacts and stable occlusion [23]. In conventional methods, articulating paper is a time-consuming, subjective, and often qualitative tool commonly used in OSSs for the determination of contact points that require adjustments. Although an OSS is widely used in clinical practice, there is a lack of understanding about the utility of an OSS for a technology that could guide occlusal adjustment [24]. In other words, the current knowledge of OSSs in treatment of bruxism has mainly depended on dentists’ professional experience. Our proposed sensor device now makes it possible to change the way we treat bruxism from a typical subjective judgment to a data-based objective approach by combining the advantages of using chips and OSSs. The modified OSS shows a similar function as the T-Scan III system (Tekscan Inc. Boston, MA USA) and allows occlusal forces to be assessed objectively and dynamically during an articulation cycle [25]. Using the device, a bruxism patient can also learn to voluntarily control abnormal grinding or clenching events based on an occlusal force detection system and prompt the immediate relaxation of the jaw muscles [18]. The ultimate goal is to reduce or eliminate the symptoms of bruxism by self-regulation without the aid of a feedback device.

### 4.3. Chip Selection

The whole sensor system integration solution was designed from scratch, considering both the hardware and firmware designs. In order to achieve real-time, dynamic monitoring of bite forces, a full-dentition occlusion detector must be worn on a patient’s upper or lower jaw dentition. When exposed to an oral environment, special requirements, such as miniature, low power consumption, and stable performance, must be considered to determine the size and the power consumption of the selected module. The main control module is the core and foundation of the entire detection system, which determines whether the entire system can detect and transmit data in a stable and accurate manner. The chips in the modified OSS have a thickness of approximately 300 μm, whereas the digital sensor of the T-scan III system is 100 μm thick [25]. The impact of chip thickness is thus a main study direction in the future.

### 4.4. Embedding Challenges

Achieving stress sensor device integration and embedding in the mouth is subject to various challenges, and the core problem has to do with how we can successfully integrate a sensor chip into an OSS and operate it normally. In conventional methods, an OSS is fabricated by a manual workflow with the polymethyl methacrylate (PMMA) material [26]. With the emergence of digital technologies, computer-aided design and computer-aided manufacturing (CAD-CAM) additive (printing) or subtractive (milling) production of OSSs with PMMA-based resins have been proposed as great alternatives [26,27,28]. Additionally, recently introduced intraoral scanners can be integrated with the described system, in order to have a direct three-dimensional (3D) reproduction of dental arches without intermediate passages. These impression systems allow for an easy interaction with CAD/CAM printers and thus a completely digital workflow, with sufficient clinical precision and greater patients’ satisfaction if compared with conventional impression methods [29]. Stress-sensing chips may experience degraded performance under high temperature and high stress. Therefore, how to effectively combine traditional materials that require high-temperature and high-stress fabrication with chips that cannot withstand high temperature and high stress is the most basic problem that must be solved. At present time, both traditional approaches and digital methods are unable to propose an optimal strategy for embedding chips into OSS resins [10,11,12]. Although the modified OSS could be accomplished by light-curing, digital technology manufacturing and comparable resin selection may be new directions for future research. Moreover, future research may consider larger samples and sufficient monitoring periods for identifying clinical reliability, validity, sensitivity, and specificity features.

### 4.5. Experiments and Future Work

One of the main novelties of this paper is using an AI algorithm for occlusal force adjustment. The input of the machine learning algorithm includes: (1) magnitude of the average occlusion force, (2) duration of the average occlusal force, (3) average contact area of occlusal forces, (4) contact points of occlusal forces, and (5) feedback of a patient pain perception level; the output of the machine learning algorithm is the level of occlusion adjustment. Therefore, the experiment results included all the inputs and the output. As well-known, machine learning algorithms need a mass of training with abundant data to obtain more accurate results. We proposed a new idea and implemented a primitive prototype in this paper. We did not obtain very large data for the machine learning algorithm. Therefore, we showed part of demonstrations of the whole system with one group of sensors and related control devices. The sampling frequency of the input signal is high enough for the bruxism frequency (the frequency of bruxism is much less than the sampling frequency). All our experiment data came from real sensors and related control devices.

The research of this concept is a complex process and requires a lot of experimental verification. In the current paper, the demonstration of the results was based on the laboratory study. We hope to share this biofeedback treatment concept and the preliminary in vitro results, which may be useful in future research of bruxism treatment. 

## 5. Conclusions

In this paper, we proposed a quantitative diagnosis and treatment framework for the treatment of bruxism using a full-dentition, intelligent occlusion stress sensor system. This study tested and verified a full-dentition, stress sensor integration and embedding approach using the proposed sandwich method combined with occlusal force data processing based on a machine learning algorithm. Experiment results showed the reasonable parameter metrics for the sensors system and demonstrated the feasibility of the scheme for effective bruxism treatment.

## Figures and Tables

**Table 1:** Reasonable parameters for the sandwich method.

Item	Parameter
Each layer of the light-cured resin	1 mm
Thickness of the piezoresistive-film sensor	0.3 mm
Light-curing time	5 min
Thickness of the OSS	2 mm

**Table 2:** The model configuration of a neural network.

Operator	Input of the Neural Network	Output of the Neural Network	Recursion Time
**Conv1d**	16	32	1
**Maxpooling**	32	32	1
**Bottleneck1d**	32	32	6
**Conv1d**	32	64	1
**Maxpooling**	64	64	1
**Bottleneck1d**	64	64	6
**Conv1d**	64	128	1
**Maxpooling**	128	128	1
**Bottleneck1d**	128	128	6
**Conv1d**	128	256	1
**Maxpooling**	256	256	1
**GAP**	256	256	1
**Fully Connected**	256	64	1
**Fully Connected**	64	3	1

## References

[1] Lobbezoo F., Jacobs R., De Laat A., Aarab G., Wetselaar P., Manfredini D. (2017). Chewing on bruxism. Diagnosis, imaging, epidemiology and aetiology. Ned. Tijdschr. Tandheelkd..

[2] Lobbezoo F., Ahlberg J., Glaros A.G., Kato T., Koyano K., Lavigne G.J., de Leeuw R., Manfredini D., Svensson P., Winocur E. (2013). Bruxism defined and graded: An international consensus. J. Oral. Rehabil..

[3] Martynowicz H., Gac P., Brzecka A., Poreba R., Wojakowska A., Mazur G., Smardz J., Wieckiewicz M. (2019). The relationship between sleep bruxism and obstructive sleep apnea based on polysomnographic findings. J. Clin. Med..

[4] Manfredini D., Restrepo C., Diaz-Serrano K., Winocur E., Lobbezoo F. (2013). Prevalence of sleep bruxism in children: A systematic review of the literature. J. Oral. Rehabil..

[5] Zhou Y., Gao J., Luo L., Wang Y. (2016). Does bruxism contribute to dental implant failure? A systematic review and meta-analysis. Clin. Implant. Dent. Relat. Res..

[6] Lobbezoo F., Ahlberg J., Raphael K.G., Wetselaar P., Glaros A.G., Kato T., Santiago V., Winocur E., De Laat A., De Leeuw R. (2018). International consensus on the assessment of bruxism: Report of a work in progress. J. Oral. Rehabil..

[7] Castroflorio T., Deregibus A., Bargellini A., Debernardi C., Manfredini D. (2014). Detection of sleep bruxism: Comparison between an electromyographic and electrocardiographic portable holter and polysomnography. J. Oral. Rehabil..

[8] Jokubauskas L., Baltrušaitytė A. (2018). Baltrusaityte, Efficacy of biofeedback therapy on sleep bruxism: A systematic review and meta-analysis. J. Oral. Rehabil..

[9] Wang L.F., Long H., Deng M., Xu H., Fang J., Fan Y., Bai D., Han X.L. (2014). Biofeedback treatment for sleep bruxism: A systematic review. Sleep Breath..

[10] Baba K., Clark G.T., Watanabe T., Ohyama T. (2003). Bruxism force detection by a piezoelectric film-based recording device in sleeping humans. J. Orofac. Pain.

[11] Nakamura H., Takaba M., Abe Y., Yoshizawa S., Suganuma T., Yoshida Y., Nakazato Y., Ono Y., Clark G.T., Baba K. (2019). Effects of a contingent vibratory stimulus delivered by an intra-oral device on sleep bruxism: A pilot study. Sleep Breath..

[12] Gu W., Yang J., Zhang F., Yin X., Wei X., Wang C. (2015). Efficacy of biofeedback therapy via a mini wireless device on sleep bruxism contrasted with occlusal splint: A pilot study. J. Biomed. Res..

[13] Macedo C.R., Silva A.B., Machado M.A., Saconato H., Prado G.F. (2007). Occlusal splints for treating sleep bruxism (tooth grinding). Cochrane Database Syst. Rev..

[14] Karakis D., Dogan A., Bek B. (2014). Evaluation of the effect of two different occlusal splints on maximum occlusal force in patients with sleep bruxism: A pilot study. J. Adv. Prosthodont..

[15] Matsumoto H., Tsukiyama Y., Kuwatsuru R., Koyano K. (2015). The effect of intermittent use of occlusal splint devices on sleep bruxism: A 4-week observation with a portable electromyographic recording device. J. Oral. Rehabil..

[16] Hannun A.Y., Rajpurkar P., Haghpanahi M., Tison G.H., Bourn C., Turakhia M.P., Ng A.Y. (2019). Cardiologist-level arrhythmia detection and classification in ambulatory electrocardiograms using a deep neural network. Nat. Med..

[17] Ravizza S., Huschto T., Adamov A., Bohm L., Busser A., Flother F.F., Hinzmann R., Konig H., McAhren S.M., Robertson D.H. (2019). Predicting the early risk of chronic kidney disease in patients with diabetes using real-world data. Nat. Med..

[18] Ilovar S., Zolger D., Castrillon E., Car J., Huckvale K. (2014). Biofeedback for treatment of awake and sleep bruxism in adults: Systematic review protocol. Syst. Rev..

[19] Sumonsiri P., Thongudomporn U., Paphangkorakit J., Premprabha T. (2019). Assessment of the relationship between masticatory performance, occlusal contact area, chewing time and cycles, and gastric emptying scintigraphy in dentate subjects. J. Oral. Rehabil..

[20] Beddis H., Pemberton M., Davies S. (2018). Sleep bruxism: An overview for clinicians. Br. Dent. J..

[21] Lobbezoo F., Davies S., van der Zaag J., van Selms M.K., Hamburger H.L., Naeije M. (2008). Principles for the management of bruxism. J. Oral. Rehabil..

[22] Shulman J. (2001). Teaching patients how to stop bruxing habits. J. Am. Dent. Assoc..

[23] Majithia I.P., Arora V., Anil Kumar S., Saxena V., Mittal M. (2015). Comparison of articulating paper markings and T Scan III recordings to evaluate occlusal force in normal and rehabilitated maxillofacial trauma patients. Med. J. Armed. Forces. India.

[24] Candirli C., Korkmaz Y.T., Celikoglu M., Altintas S.H., Coskun U., Memis S. (2016). Dentists’ knowledge of occlusal splint therapy for bruxism and temporomandibular joint disorders. Niger. J. Clin. Pract..

[25] Nota A., Tecco S., Cioffi C., Beraldi A., Padulo J., Baldini A. (2019). Occlusion time analysis in military pilots affected by bruxism. Sci. Rep..

[26] Huettig F., Kustermann A., Kuscu E., Geis-Gerstorfer J., Spintzyk S. (2017). Polishability and wear resistance of splint material for oral appliances produced with conventional, subtractive, and additive manufacturing. J. Mech. Behav. Biomed. Mater..

[27] Berntsen C., Kleven M., Heian M., Hjortsjö C. (2018). Clinical comparison of conventional and additive manufactured stabilization splints. Acta Biomater. Odontol. Scand..

[28] Dedem P., Türp J.C. (2016). Digital Michigan splint—from intraoral scanning to plasterless manufacturing. Int. J. Comput. Dent..

[29] Sfondrini M.F., Gandini P., Malfatto M., Di Corato F., Trovati F., Scribante A. (2018). Computerized Casts for Orthodontic Purpose Using Powder-Free Intraoral Scanners: Accuracy, Execution Time, and Patient Feedback. Biomed. Res. Int..

